# Brain-Derived Steroids, Behavior and Endocrine Conflicts Across Life History Stages in Birds: A Perspective

**DOI:** 10.3389/fendo.2018.00270

**Published:** 2018-06-18

**Authors:** John C. Wingfield, Douglas W. Wacker, George E. Bentley, Kazuyoshi Tsutsui

**Affiliations:** ^1^Department of Neurobiology Physiology and Behavior, University of California, Davis, Davis, CA, United States; ^2^Division of Biological Sciences, School of STEM, University of Washington Bothell, Bothell, WA, United States; ^3^Department of Integrative Biology, Helen Wills Neuroscience Institute, University of California, Berkeley, Berkeley, CA, United States; ^4^Department of Biology, Waseda University, Shinjuku, Japan

**Keywords:** territorial aggression, life history stages, aromatase, dehydropepiandrosterone, 7α-hydroxypregnenolone, locomotor activity, neurosteroid

## Abstract

Biological steroids were traditionally thought to be synthesized exclusively by the adrenal glands and gonads. Recent decades have seen the discovery of neurosteroid production that acts locally within the central nervous system to affect physiology and behavior. These actions include, for example, regulation of aggressive behavior, such as territoriality, and locomotor movement associated with migration. Important questions then arose as to how and why neurosteroid production evolved and why similar steroids of peripheral origin do not always fulfill these central roles? Investigations of free-living vertebrates suggest that synthesis and action of bioactive steroids within the brain may have evolved to regulate expression of specific behavior in different life history stages. Synthesis and secretion of these hormones from peripheral glands is broadcast throughout the organism *via* the blood stream. While widespread, general actions of steroids released into the blood might be relevant for regulation of morphological, physiological, and behavioral traits in one life history stage, such hormonal release may not be appropriate in other stages. Specific and localized production of bioactive steroids in the brain, but not released into the periphery, could be a way to avoid such conflicts. Two examples are highlighted. First, we compare the control of territorial aggression of songbirds in the breeding season under the influence of gonadal steroids with autumnal (non-breeding) territoriality regulated by sex steroid production in the brain either from circulating precursors such as dehydroepiandrosterone or local central production of sex steroids *de novo* from cholesterol. Second, we outline the production of 7α-hydroxypregnenolone within the brain that appears to affect locomotor behavior in several contexts. Local production of these steroids in the brain may provide specific regulation of behavioral traits throughout the year and independently of life history stage.

## Introduction

The life cycles of animals consist of life history stages such as breeding, migration, molt, and non-breeding expressed at appropriate times of year and with specific durations matched to seasonal change [e.g., Ref. (1, 2)]. Each stage has a unique suite of sub-stages in which animals exhibit morphological, physiological, and behavioral adaptations to changing environmental and social conditions. Some traits may be expressed across multiple life history stages. However, the neuroendocrine and peripheral endocrine regulation of those traits in one stage might be different in another stage. Examples include the regulation of territorial aggression across breeding and non-breeding seasons (3–5), the control of locomotor activity in seasonal migration and facultative movements to avoid perturbations of the environment [e.g., Ref. (6–8)].

**Figure 1:**
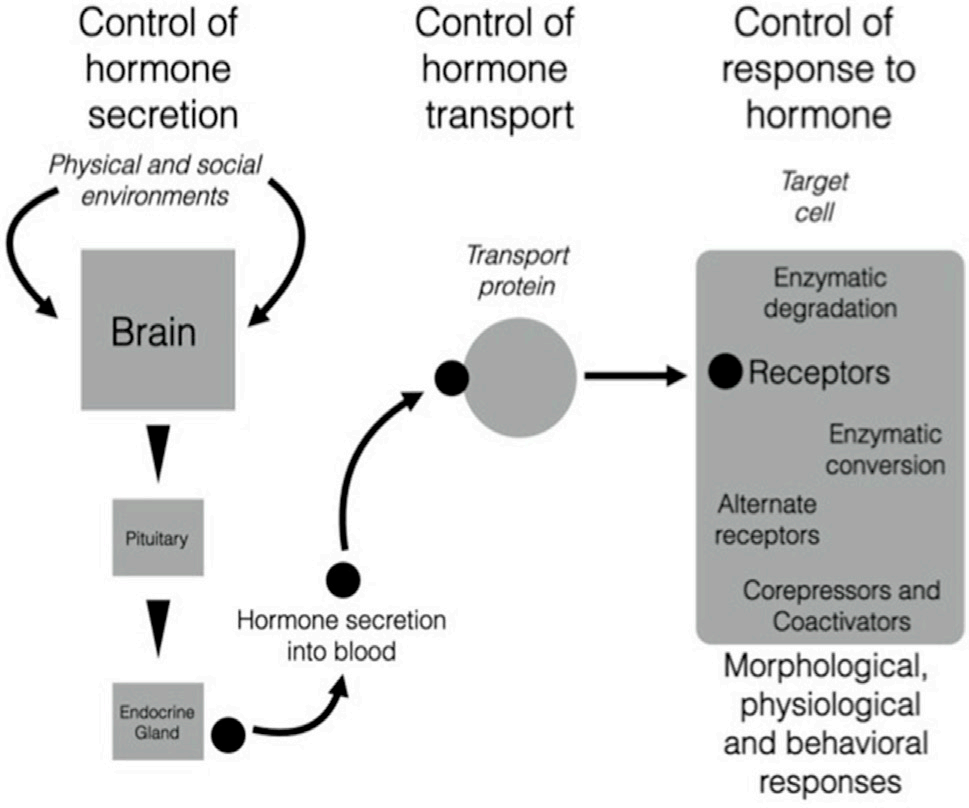
There are three components of the action of hormones that can be regulated in diverse ways. The left-hand part of the figure shows the triggering of neural, neuroendocrine, and endocrine cascades that result in production of a highly specific secreted signal. The central part of the figure shows that after secretion into the blood, many hormones (particularly steroids, thyroid hormones, and some peptides) are transported by carrier proteins. Once the hormonal signal reaches the target cell (e.g., a neuron in the brain), it can act in various ways by binding to membrane or nuclear receptors that trigger extremely complex actions in a cell. Note that all three components are sites of regulation that could change daily or seasonally, or vary among individuals, populations, sex, or in response to unique experience of the environment. It is possible that this scheme is highly conserved across vertebrates and that different species modulate different components and still achieve the same behavioral and physiological end points. See text for more details. From Wingfield (10), courtesy of the American Ornithologist’s Union and Wingfield (11), courtesy of Elsevier.

**Figure 2:**
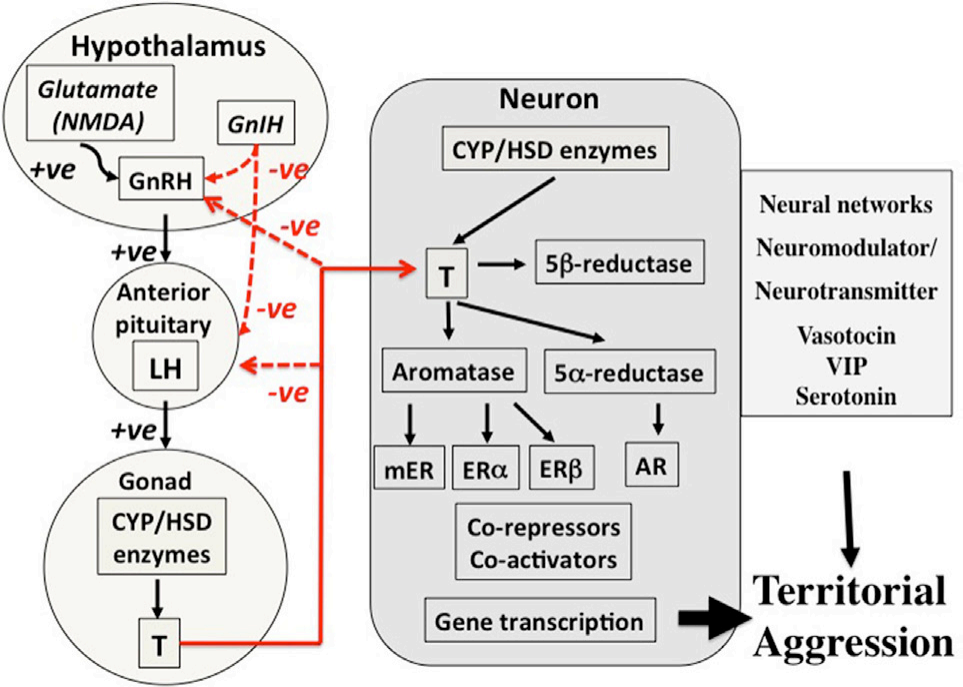
Control mechanisms for territorial aggression in birds in spring. There are three major components to control mechanisms. First, the regulation of hormone secretion from the hypothalamo–pituitary–gonad axis (left part of the figure), transport of hormones such as testosterone in the blood (lines in red), and the mechanisms associated with action of the hormone in the target cell, in this case a neuron in the brain (central part of the figure). The net result is regulation of territorial aggression by neural networks (right-hand part of the figure). This three-part system of control of hormone secretion, transport, and effects on target organs is an important concept because it offers many points of potential regulation. The secretion component on the left summarizes how sensory information, in this case social, in transduced through neurotransmitter, e.g., glutamate (NMDA), and neuroendocrine secretions such as gonadotropin-releasing hormone (GnRH) and gonadotropin-inhibiting hormone (GnIH) into release of gonadotropins luteinizing hormone (LH) and follicle-stimulating hormone (not shown) from the anterior pituitary into the blood. LH travels to the gonad where it acts on cells that express steroidogenic enzymes to stimulate secretion of the sex steroid hormone testosterone (T) that is in turn released into the blood. Local actions of T in the testis include regulation of spermatogenesis, but it is also released into the blood in many avian species during breeding. Among many actions of T are effects on territorial aggression, and negative feedback on neuroendocrine and pituitary secretions (red lines). In birds, T circulates bound weakly to corticosteroid-binding globulin before entering target neurons involved in the expression of territorial aggression (center of figure). Once T has entered a target neuron, it has four potential fates. First, it can bind directly to the androgen receptor (AR), a member of the type 1 genomic receptors that become gene transcription factors once they are bound to T. Second, T can be converted to estradiol (E2) by the enzyme aromatase. E2 can then bind to either estrogen receptor alpha (ERα) or estrogen receptor beta (ERβ) both of which are genomic receptors that regulate gene transcription, but different genes from those regulated by AR. Third, T can be converted to 5α-dihydrotestosterone that also binds to AR and cannot be aromatized thus enhancing the AR gene transcription pathway. Fourth, T can be converted to 5β-dihydrotestosterone that binds to no known receptors and also cannot be aromatized indicating a deactivation shunt. The possibility of a rapid acting membrane receptor (non-genomic) should also be considered. A complex system of co-repressors and co-activators of genomic steroid receptor action are also known. Furthermore, it is now known that many neurons in vertebrate brains express all the enzymes required to synthesize T and E2 *de novo* from cholesterol or from circulating inert steroid hormone precursors such as dehydroepiandrosterone, androstenedione, and progesterone. The end result is regulatory action on neural networks that regulate expression of territorial aggression. Several neurotransmitters and neuromodulators such as arginine vasotocin, vasoactive intestinal peptide (VIP), and serotonin as also involved at this level. Evidence suggests that the basic secretory, transport, and action mechanisms are conserved across vertebrates. However, it is also clear that there are very many points at which the expression of territorial aggression may be affected indicating almost limitless combinations of possible regulatory mechanisms. From Wingfield (10), courtesy of the American Ornithologist’s Union and Wingfield (11), courtesy of Elsevier.

There is growing evidence that the neuroendocrine and endocrine cascades involved in regulation of morphology, physiology, and behavior across different life history stages can be altered at many different levels [e.g., Ref. (9); Figure 1]. Localized regulatory mechanisms in one life history stage allow trait modulation without the effects associated with signals that act throughout the organism such as hormones secreted into the bloodstream acting on multiple tissues. Thus, conflict between the need for the expression of a particular trait that can be regulated by a particular hormone and the inappropriate co-regulation of other physiological or behavioral responses to that hormone at the wrong season can be avoided. These regulatory processes can be organized into three major categories [see Figure 1 (10, 11)]:

1. Secretion—cascades of hormone secretion [e.g., the hypothalamo–pituitary–gonad (HPG) axis, Figure 2] in which hormones are broadcast throughout the organism’s bloodstream and have as many potential targets as cells or tissues that possess receptors for that hormone.
2. Transport—transport systems by which hormones are transported in the blood [e.g., corticosteroid-binding globulins (CBG)], or are selectively taken up by organs and tissues *via* other cellular transport proteins (e.g., the blood–brain barrier).
3. Response—regulated responses of target cells to, and fine-tuned synthesis/availability of, paracrine, neuroendocrine, and endocrine secretions that can facilitate appropriate life history stage changes. These processes may involve alterations to receptor expression within a target cell, actions of metabolizing enzymes that can promote or deactivate a hormone (e.g., steroid metabolizing enzymes in many tissues that regulate local substrate availability for endocrine signaling systems), and a complex system of proteins that can act as enhancers or inhibitors of gene expression. It is possible to take this further downstream to protein synthesis, packaging, and actions, but these are beyond the scope of this paper.

An example of a secretion–transport–response system is the production and action of sex steroids (Figure 2), in which the HPG cascade leading to testosterone secretion has multiple levels of possible regulation. Many species of vertebrates have highly specific androgen-binding proteins in the blood, including sex hormone-binding globulin, which is absent in birds, as well as CBG, which binds both glucocorticoids and sex steroids (12–14). The CBG gene has recently been identified in birds (15) and will allow future studies on CBG regulation. Target cell responses can be regulated in various ways, including the transformation of testosterone to estradiol and thus activation of a different array of receptor types [Figure 2 (16, 17)]. Also, testosterone can be deactivated by 5β-reductase to 5β-dihydrotestosterone that is generally regarded as inert and does not bind to androgen receptors (ARs) [Figure 2 (18–20)]. Note that peripheral secretion of sex steroids (left-hand side of Figures 1 and 2) results in broadcast of hormones to the entire organism through the blood stream, whereas actions at target cells (right-hand part of the Figures 1 and 2) allows local adjustment of the responses to a hormone. Steroids produced within the brain also allow paracrine actions at very localized sites without the additional effects on general morphological, physiological, and behavioral responses that occur when the hormone is broadcast through the blood stream [e.g., Ref. (11)]. These flexible points of regulation allow for the fine-tuning of trait expression to meet the specific demands of a stimulus, while keeping the animal within the necessary constraints of its current life history stage.

The secretion–transport–response regulatory model allows for the progressive localization of the effects of blood borne hormones (Figure 2; Figure S1 in Supplementary Material). Secretion cascades are generally broadcast to the whole organism whereas transport and especially target cell responses can be tailored to specific situations, environmental change, and life history stage. Environmental cues emanating from changing conditions can have direct effects on the secretion–transport–response system (e.g., temperature-related changes in hormone metabolism) or, more commonly, can be signalized *via* neural perception and transduction [Figure S1 in Supplementary Material, see Ref. (21)]. Mechanisms by which environmental cues can affect transport mechanisms and specific target cell responses remain much less well known. For example, it appears that the testes of zebra finches, *Taenopygia guttata*, can integrate and respond to cues of stress directly, rather than relying on a hormonal cascade initiated by the brain (22, 23), but it is not known exactly why the brain is bypassed in this instance. The evolutionary origins of the endocrine axes have seemingly resulted in a remarkable flexibility of response of different tissues (24).

### Neurosteroids in Vertebrate Brains

Sex steroids have multiple peripheral and central actions on physiological traits. Sex steroids are not only produced solely by gonads and adrenal glands, but also by the brain [e.g., Ref. (5, 9, 25–31)]. In seasonally breeding vertebrates, neurally derived sex steroids produced in the non-breeding season do not influence reproductive traits *per se*, as very little sex steroid from this source reaches the blood stream and such neurosteroids are synthesized and act in a paracrine fashion in localized areas of the brain (32). Still, neurosteroids can have numerous effects on behavioral traits such as locomotor activity, aggression, reproduction, body temperature, and blood pressure (9, 29, 33–38). In addition, steroids in specific brain nuclei show seasonal changes in concentration, independently of circulating steroids [e.g., Ref. (39)]. These neurosteroids then act in a variety of brain regions to facilitate appropriate behavior for each life history stage (3, 28, 30, 40, 41).

The seasonal regulation of neurosteroid expression is not restricted to sex steroids. In a study on Japanese newts, *Cynops pyrrhogaster*, concentrations of pregnenolone and progesterone in the brain were higher in animals exposed to long spring-like vs. short winter-like day lengths, whereas experimental manipulation of environmental temperature had no effect (28). The newt brain expresses enzymes that synthesize 7α-hydroxypregnenolone *de novo* from cholesterol, and this neurosteroid activates locomotor activity associated with spring breeding. Moreover, the effect of 7α-hydroxypregnenolone on locomotor activity was blocked by dopamine D2-like receptor antagonists (27).

## Two Examples of Flexibility in Steroid Mechanisms of Action

We will focus next on two examples in which local production and paracrine actions of steroids in the brain can have major impacts on control of behavior and potentially avoid evolutionary costs of peripheral endocrine secretions at the wrong time.

### Androgen/Estrogen Control of Avian Territorial Aggression Across Life History Stages

Although male territorial aggression was classically thought to be associated with circulating testosterone, work over the last 30 years suggests that the neural sensitivity to sex steroids, and the synthesis and action of both androgens and estrogens can modulate such behavior (32, 38, 42, 43). These are particularly important findings with regards to seasonal animals that express aggression during non-breeding life history stages when high circulating testosterone levels would likely lead to inappropriately timed reproductive behavior. For example, the Pacific Northwest subspecies of the song sparrow (*Melospiza melodia morphna*) shows high levels of aggressive territoriality and circulating testosterone during its breeding life history stage, low levels of testosterone and aggression during the autumnal molt, and high levels of aggression, but very low levels of circulating testosterone during the post-molt, non-breeding period (44). Our three-point secretion–transport–response regulatory model helps to explain how such behavioral expression is achieved across these different life history stages.

Secretion and production of sex steroids that modulate territorial aggression in the song sparrow change seasonally. For example, there is evidence for local production of testosterone and 17β-estradiol in the brain, both *de novo* from cholesterol and from circulating precursors (32, 33, 39). An example of the latter is dehydroepiandrosterone (DHEA), which is released by the adrenal glands, liver, and other peripheral organs, taken up by brain regions, and converted locally to more bioactive steroids (39). Neural steroid production allows for animals to retain actions of sex steroids in regions of the brain relevant to aggression, while avoiding the complication and inappropriate activation of morphology, physiology, and behavior associated with reproduction at the wrong time of year (45). Measurement of circulating DHEA indicates seasonal changes associated with territorial behavior in different life history stages in some species [e.g., Ref. (3, 19)] but not others [e.g., Ref. (46)]. In the song sparrow, circulating DHEA levels are highest during breeding and non-breeding when aggression is highest and lower during the molt, when aggression is also low, suggesting that circulating DHEA acts to promote life history appropriate aggression in this species (39).

The peripheral transport of DHEA and its movement into the brain, especially in birds, is not well characterized. In humans and some other vertebrates, DHEA travels in a hydrophilic sulfated form, DHEAS [e.g., Ref. (47)]. However, in birds, including the song sparrow, DHEA circulates primarily in its non-sulfated form [e.g., Ref. (39)]. Once DHEA arrives at its targets in the brain, it is possible that it has direct effects *via* ERβ, sigma-1, and/or GABAa receptor activation (48–50), though the affinity of DHEA for these receptors can be very low and more work is needed to assess the physiological and behavioral relevance of these possibilities. It is perhaps more likely that DHEA exerts the bulk of its effects on aggression *via* local conversion to testosterone, 5α-dihydrotestosterone, and 17β-estradiol, and subsequent activation of androgen and estrogen receptors.

Although circulating DHEA levels are equally high in breeding and non-breeding song sparrows, its local conversion to testosterone/estrogen in the brain may be more important during the non-breeding life history sub-stage (51). The activity of 3β-hydroxysteroid dehydrogenase/Δ^5^-Δ^4^-isomerase (3β-HSD), the enzyme that catalyzes transformation of DHEA to androstenedione, is higher during non-breeding vs. breeding in many brain regions that comprise neural networks underlying social behavior (30). Furthermore, DHEA is rapidly metabolized in such brain regions in dominant male song sparrows during aggressive interactions (42). These same interactions do not alter plasma DHEA levels. Evidence suggests that DHEA is eventually converted to 17β-estradiol to facilitate non-breeding aggression in this species.

Fadrozole, an inhibitor of aromatase, the enzyme that catalyzes the transformation of androgens to estrogens, reduces non-breeding, but not breeding aggression in song sparrows (20). That the precursor, DHEA might play a more prominent role in steroid-dependent, non-breeding aggression makes intuitive sense, as elevated circulating testosterone likely provides ample substrate to brain regions modulating aggression during the breeding period. Furthermore, castration of male song sparrows had no effect on territorial aggression in autumn suggesting that gonadal sources of androgen were not essential for expression of territoriality (52). These findings strengthen the hypothesis that circulating steroid hormone precursors such as DHEA and/or local production of sex steroids in the brain are requisite for expression of aggression in the non-breeding season in some species.

Estrogen synthesis can also be modified to facilitate appropriate behavioral responses. Changes in estrogen synthesis can occur in response to immediate environmental or social cues. For example, aromatase can be rapidly inactivated *via* phosphorylation (53), which may help to quickly alter behavior when an immediate context warrants it, though much more work is needed to address the behavioral relevance of these acute changes (16, 54). Estrogen synthesis can also change seasonally, potentially facilitating life history stage appropriate responses. In the male song sparrow, aromatase activity is highest in the ventromedial telencephalon, home to the medial amygdala, during breeding and non-breeding life history stages, when aggression is also at its height (3). The expression of aromatase mRNA also changes seasonally in multiple brain regions associated with the control of social behavior in the male song sparrow (5). Aromatase expression in the ventromedial nucleus of the hypothalamus (VMH), a brain region known to be involved in aggression-seeking behavior in rats (55), is equally high during breeding and non-breeding, when aggression is also highest, suggesting that the VMH may also mediate aggressive responses in birds (5). Aromatase expression in the preoptic area (POA), a brain region long known to be involved in the regulation of reproductive behavior and physiology, is both elevated during breeding and increased by administration of exogenous DHEA in male song sparrows, suggesting that this androgen/estrogen precursor may mediate different effects in different life history stages (56).

Androgen and estrogen receptor sensitivity may also be modulated to promote life history stage appropriate behavior. For example, neural AR mRNA expression changes seasonally in male song sparrows, with higher levels in the POA, the periventricular nucleus of the medial striatum (pvMSt), and paraHVC, a region of the song control system, during breeding (5). Exogenously administered DHEA increases AR expression in many of the same regions, again suggesting a role for this hormone in the seasonal modulation of social behaviors in this species (56). Current evidence suggests that DHEA, its transport to the brain from peripheral tissues, and its local conversion to androgens and estrogens helps promote life history appropriate behaviors, including seasonal aggression, in the male song sparrow. Adjustments of AR and aromatase gene expression have also been associated with aggressive responses to simulated territorial intrusions in dark-eyed juncos, *Junco hyemalis* (43), and differences in estrogen receptor alpha (ERα) expression are associated morph-specific variation in aggressive song in white-throated sparrows, *Zonotrichia albicollis* (57). In juncos, there were no changes in ERα expression, however (58), highlighting the importance of considering species-specific differences when assessing steroid responses relating to avian territorial aggression. Changes in sex steroid receptors in specific areas of the brain appear to be a widespread mechanism by which the sensitivity of neural tissue is regulated [e.g., Ref. (59, 60)].

### Potential Roles of Neurosteroids on Animal Movement Including Migration

A second example of how the secretion–transport–response regulatory model approach informs hypotheses about how potential conflicts of steroid control of life history stages in different contexts might be avoided, involves the role of the neurosteroid 7α-hydroxypregnenolone in regulation of locomotor activity. Regular avian migrations (e.g., spring and autumn) have well-established endocrine control mechanisms [e.g., Ref. (7, 61)]. However, control mechanisms associated with specific environmental cues in one migration life history stage may have conflicts of action for the other. For example, spring migration toward a breeding area occurs during increasing day length but decreasing temperature and trophic resources as animals move north. By contrast, autumn migration toward a wintering area occurs during decreasing day length but temperatures tend to be higher as the animals move south and trophic resources are good, even increasing [e.g., Ref. (6)]. It is likely that these cues are differently transduced and integrated across these life history stages, even though the resulting migratory behaviors are similar in appearance. Furthermore, other types of animal movements such as facultative movements in response to local conditions may be different again (8). Nevertheless, common mechanisms may exist at fundamental neural levels across life history stages and facultative contexts, and there is preliminary evidence that the paracrine actions of 7α-hydroxypregnenolone may play a key role.

7α-Hydroxypregnenolone has been shown to affect locomotor behavior in newts, fish, and quail [e.g., Ref. (27, 34, 36, 40)]. Prolactin mediates 7α-hydroxypregnenolone action on spring migration in Japanese newts (62) and during upstream migration in chum salmon, *Oncorhynchus keta*, possibly *via* actions on dopaminergic neurons in the magnocellular preoptic nucleus (63). There are also strong circadian effects on 7α-hydroxypregnenolone that are possibly mediated by melatonin (36, 64), setting the stage for circadian control of locomotor behavior.

**Figure 3:**
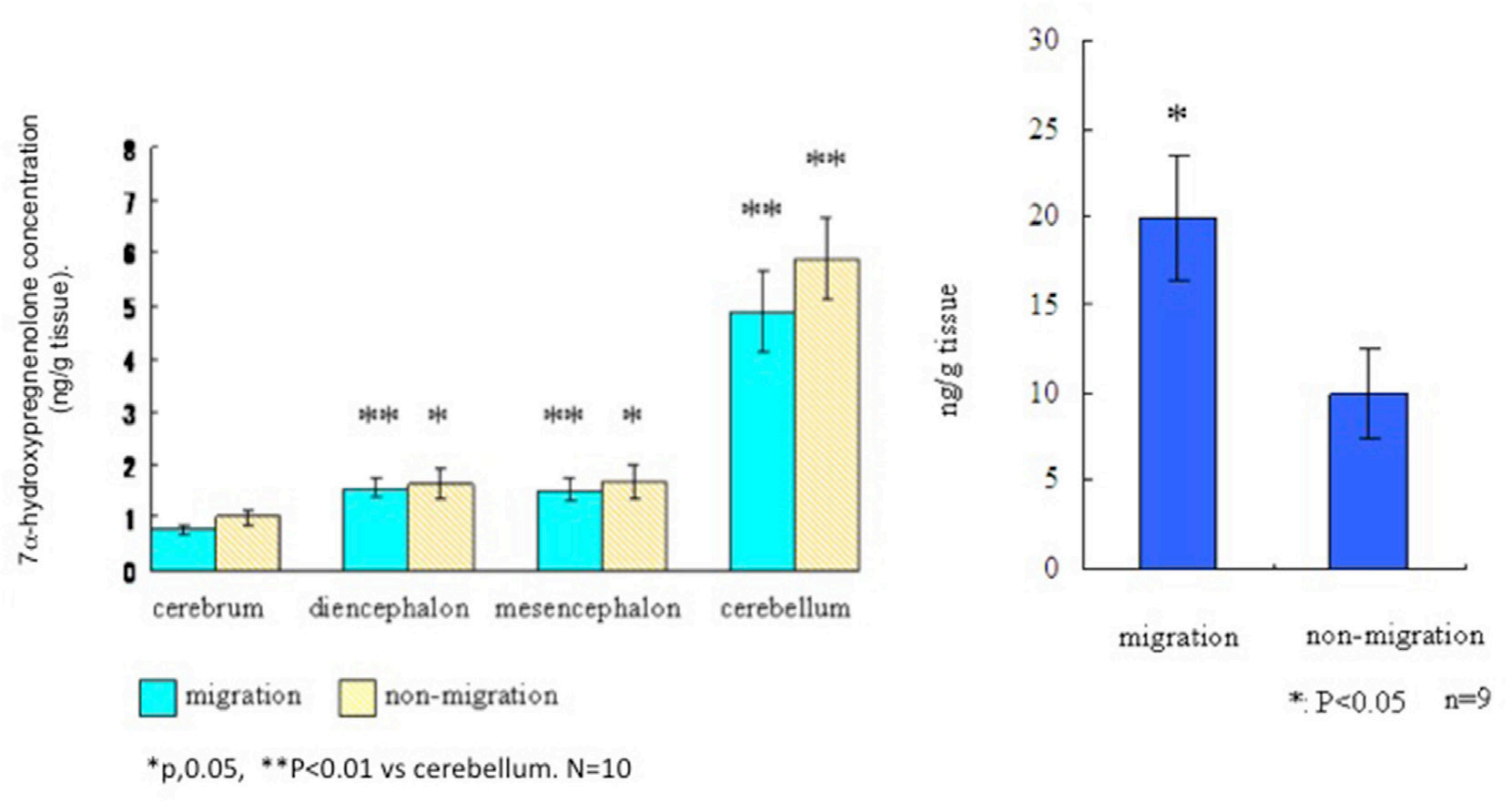
Left panel. Synthesis of 7α-hydroxypregnenolone in different brain regions of the white-crowned sparrow, *Zonotrichia leucophrys gambelii*. Right panel. Whole brain concentrations of 7α-hydroxypregnenolone in white-crowned sparrows, in migratory and non-migratory life history stages.

To assess the distribution and actions of 7α-hydroxypregnenolone in a migratory songbird, the white-crowned sparrow, *Zonotrichia leucophrys gambelii* (see Presentation S1 in Supplementary Material for methods and details), we collected brains from photostimulated birds that were in the spring migratory life history stage and divided them into regions. Left and right hemispheres were analyzed for differences in 7α-hydroxypregnenolone synthesis and concentrations, respectively. We also collected brains from birds in the non-migratory stage and analyzed 7α-hydroxypregnenolone synthesis separately in the cerebrum, diencephalon, mesencephalon, and cerebellum. There were no significant differences in 7α-hydroxypregnenolone synthesis in any brain region between the “migratory phase” and “non-migratory phase” (Figure 3). However, 7α-hydroxypregnenolone synthesis in the cerebellum was higher than those in other brain regions in both males and females and regardless of migratory and non-migratory states (Figure 3; Figure S2 in Supplementary Material).

By GC-Ms analysis for 7α-hydroxypregnenolone concentration, we combined different brain regions (cerebrum + diencephalon + mesencephalon + cerebellum) due to the small tissue amounts involved. Results from this analysis suggest that 7α-hydroxypregnenolone concentration during the spring migratory phase is higher than that during the non-migration phase (Figure 3, right-hand panel). Note also that white-crowned sparrows in the vernal migratory stage exhibited very high levels of nocturnal migratory restlessness (*Zugunruhe*) compared with photorefractory birds that were molting and not migrating (Figure S3 in Supplementary Material). A higher concentration of 7α-hydroxypregnenolone during the migratory stage may depend on the amount of its precursor pregnenolone. An analysis of P450scc producing pregnenolone may be required in order to fully elucidate the dynamics of 7α-hydroxypregnenolone synthesis during this life history stage.

To determine central behavioral effects of 7α-hydroxypregnenolone infused into the third ventricle of white-crowned sparrows, birds in the molt life history stage were used, i.e., birds not showing migratory behavior (Figure S4 in Supplementary Material). Birds were then video-recorded for 30 min post-infusion, but most of the effects occurred in the first 10 min. 7α-Hydroxypregnenolone significantly inhibited perch-hopping behavior at a dose of 200 ng in 2 μl of vehicle in molting birds, but there was a great deal of variance in the data (top panel of Figure S4 in Supplementary Material). Overall, there was a decrease in perch-hops of approximately 30% compared with activity following saline infusions in the same birds (Figure S4 in Supplementary Material). There was approximately a 90% decrease in some individuals. In a follow-up study, infusion of 7α-hydroxypregnenolone at high and low doses into white-crowned sparrows had no effect on locomotor behavior during the spring migration life history stage (lower panel, Figure S4 in Supplementary Material). These data suggest seasonal and state dependent differences in actions of neurosteroids. Further studies attempting to block the actions of 7α-hydroxypregnenolone in spring and autumn migration will be critical.

## Perspective

The regulation of morphological, physiological, and behavioral traits specific to life history stages is mediated through endocrine, neuroendocrine, and paracrine secretions. When a specific trait may be expressed in more than one life history stage, the potential for conflict and decreased fitness arises, and the regulation of that trait may differ across those stages. In other words, hormonal regulation appropriate for one life history stage may not be suitable for another. As mentioned earlier, sex steroids of gonadal origin that affect territorial aggression in the breeding season are dependent on the HPG axis and are broadcast throughout an organism *via* the blood stream. In a non-breeding life history stage, such peripheral secretion of sex steroids would also activate morphological, physiological, and behavioral responses that would be inappropriate for that life history stage. Instead, local production of sex steroids in specific areas of the brain regulate territorial aggression during non-breeding, thereby helping to avoid the costs of action of gonadal sources of sex steroids in peripheral tissues.

In other cases, production of neurosteroids in localized, specific, brain regions may regulate behavioral and physiological traits that are expressed throughout the life cycle, and also on facultative bases. The role of 7α-hydroxypregnenolone may fit this scenario, especially in relation to its actions on locomotor activity. Although 7α-hydroxypregnenolone levels in the brain were higher in white-crowned sparrows in migratory vs. non-migratory life history stages (Figure 3), the effects of central infusion are difficult to interpret (Figure S4 in Supplementary Material). This may have been because birds were already showing maximum migratory activity, or that other regions of the brain may be important for regulation of migratory behavior. Nonetheless, future investigations will be needed to determine how 7α-hydroxypregnenolone might be important for regulation of migration, and whether this regulation changes across the two migratory life history stages (vernal and autumnal migrations) in the white-crowned sparrow.

Haraguchi et al. (65) have shown that 7α-hydroxyprogesterone can increase locomotor activity following restraint stress in newts, and, along with elevated corticosterone, may regulate behavioral responses to environmental perturbations. The possibility that this neurosteroid is involved in the rapid regulation of facultative movements associated with environmental perturbations during all life history stages is an exciting hypothesis. Facultative movements could occur at any time as part of the corticosteroid-induced emergency life history stage, for example, in response to a storm or increased predator pressure [e.g., Ref. (66, 67)]. Facultative movements would need to cease once suitable alternate habitat is found for the individual to survive the perturbation. 7α-hydroxypregnenolone is one neural paracrine signal that may be involved in both seasonal and facultative behavioral changes. It may also play a role in suppressing activity in molting white-crowned sparrows.

How hormone and neurohormone secretion, transport, and response are integrated to achieve the appropriate morphological, physiological, and behavioral response to life history stage and environmental and social changes requires further integrated studies. Coordination of the biological actions of these newly discovered autocrine and paracrine actions in the brain especially need more investigation in relation to life history stage and other environmental contexts. Understanding each organism’s physiological and behavioral requirements across life history stages will allow us to identify the endocrine conflicts each faces as their requirements change. This has the potential to enlighten us as to how novel autocrine and paracrine actions within the brain and other tissues are coordinated and provide insights into the selection pressures that have driven the evolution of these systems.

## Author Contributions

JW wrote the original review and all other authors contributed equally to edits, addition of relevant references, and conceptual development.

## Conflict of Interest Statement

The authors declare that the research was conducted in the absence of any commercial or financial relationships that could be construed as a potential conflict of interest. The reviewer WG declared a past co-authorship with the author JW to the handling Editor.
