# Supplementary material for: Brain-Derived Steroids, Behavior and Endocrine Conflicts Across Life History Stages in Birds: A Perspective
Source: Front Endocrinol (Lausanne). 2018 Jun 18;9:270. doi: 10.3389/fendo.2018.00270 (PMC6015890; doi:10.3389/fendo.2018.00270)

Supplementary Materials.

Methods for determining 7a-hydroxyprenolone synthesis and concentrations (after Matsanaga et al., 2004; Tsutsui et al., 2008; Haraguchi et al., 2015).

White-crowned sparrows were anesthetized with isofurane and then decapitated. Brains were removed immediately and frozen on dry and stored at -80°C. On thawing, whole brain or sub-divided brain regions were homogenized. Each homogenate (40 mg of tissue each) was incubated with tritiated pregnenolone for 20 min at 40°C, steroids were then extracted and analyzed by High Performance Liquid Chromatography (HPLC). Concentration of endogenous 7α-hydroxypregnenolone were measured in different brain regions by GC-MS analysis as described by Matsunaga et al. (2004). In each brain region steroids were extracted using solid phase C18 columns. Whole brain was homogenized in methanol/H2O (75:25, v/v; 1 ml) on ice. After centrifugation at 3000 g for 5 min, supernatants were diluted to a final concentration of 5% methanol. Whole brain homogenates were then extracted with C18 columns equilibrated with methanol and methanol/H2O (5:95, v/v). Samples were applied to the column, and steroid fractions eluted with methanol and evaporated to dryness. Each sample was then applied to a GC-MS system as described above. The internal standard [17,21,21,21- 2H-pregnenolone was prepared as described by Matsunaga et al. (2004). In brief, [17,21,21,21- 2H-pregnenolone was synthesized from the unlabeled pregnenolone for the internal standard. Pregnenolone (100 mg) was subjected to a reflux exchange reaction in a 35% solution of 2HCl (99% 2H, 50 ul) in CH3CH2O2H (99% 2H, 3.3 ml) overnight. The final mixture was evaporated to dryness and the exchange reaction was repeated as above. Steroid products were crystallized twice from aqueous ethanol. Synthetic 2H-labeled steroid then was isolated as a single peak on a HPLC column before use as an internal standard.

Statistics.

Methods for infusion of 7α-hydroxy-pregnenolone into the third ventricle of white-crowned sparrows.

Supplementary Figures.

Figure S.1. Secretion-transport-response, a three part system of potential regulatory mechanisms. Secretion cascades are broadcast to the whole organism whereas transport and especially target cell responses can be customized such that an animal responds appropriately to environmental and social change. Environmental and social cues emanating from changing conditions can have direct effects on the secretion – transport – response system or can be signalized via neural perception and transduction (see Wingfield and Mukai, 2009).

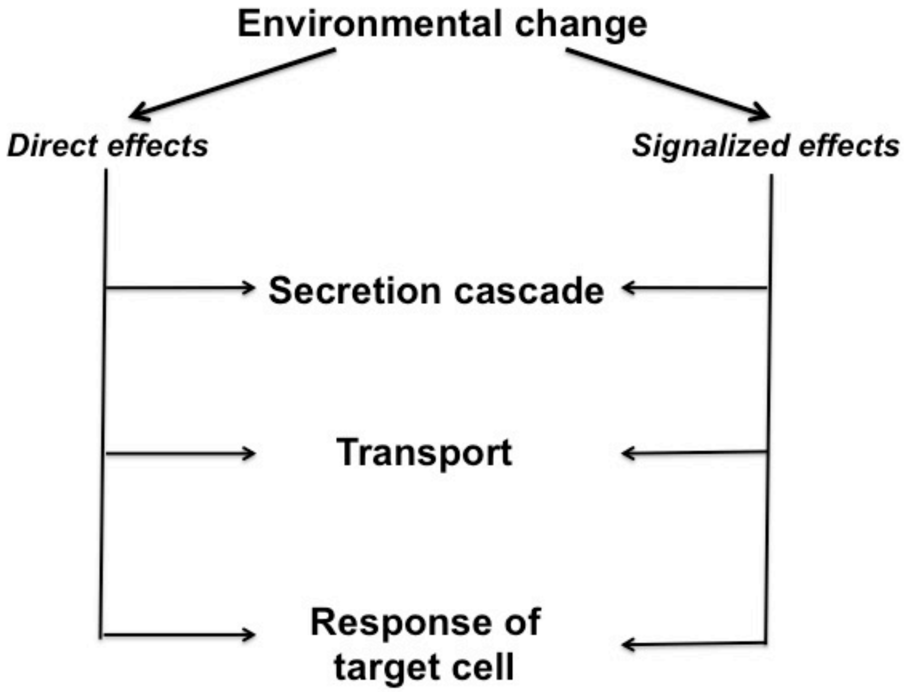

Figure S.2. Biosynthesis of 7α-hydroxypregnenolone in brain regions of the white-crowned sparrow, *Zonotrichia leucophrys gambelii* in relation to migratory and non-migratory life history stages and to sex.

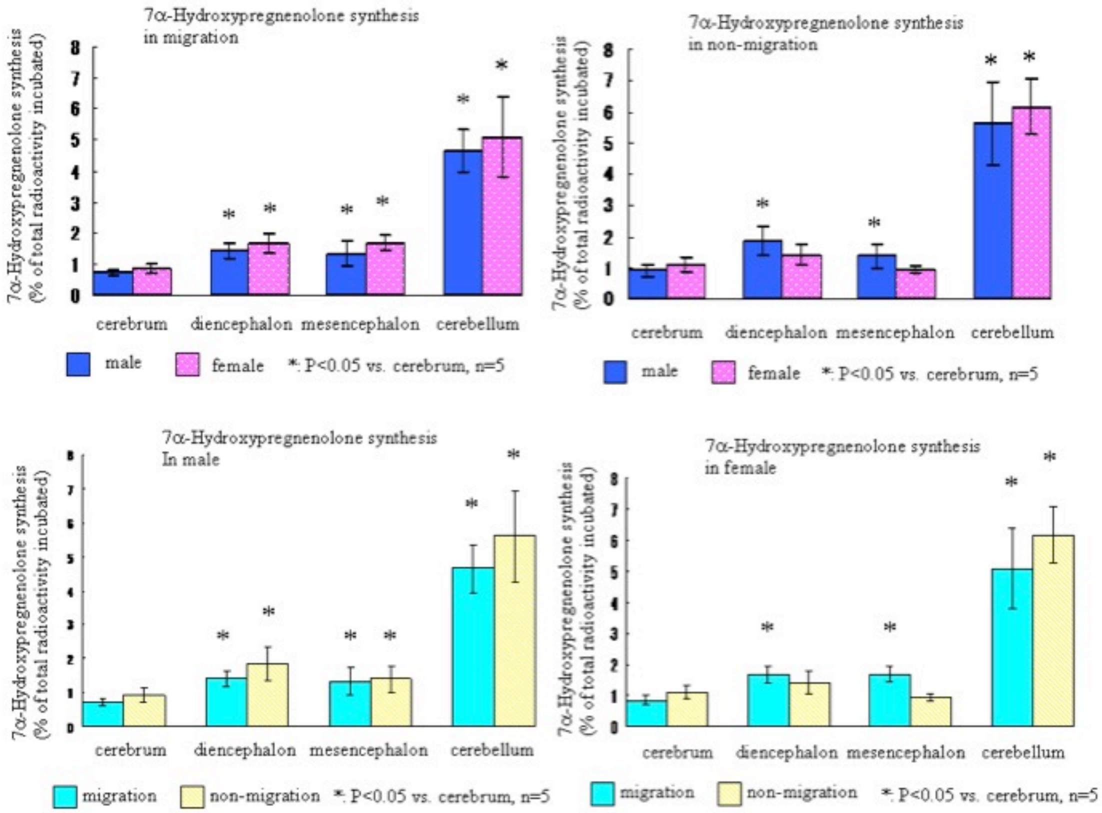

Figure S.3. Daily and nocturnal perch hopping activity of white-crowned sparrows measured as the average number of beam breaks (of an infra-red detector, BB/min) in relation to hours of the day. Data show spring migratory activity after exposure to long days (top panel). Zug = *zugunruhe* – migratory restlessness). Compare this to the lack of migratory-like behavior at night in molting birds (bottom panel, phrefr = photorefractory birds that have terminated the breeding life history stage). In both panels the horizontal black bar represents the dark phase of the daily photoperiod.

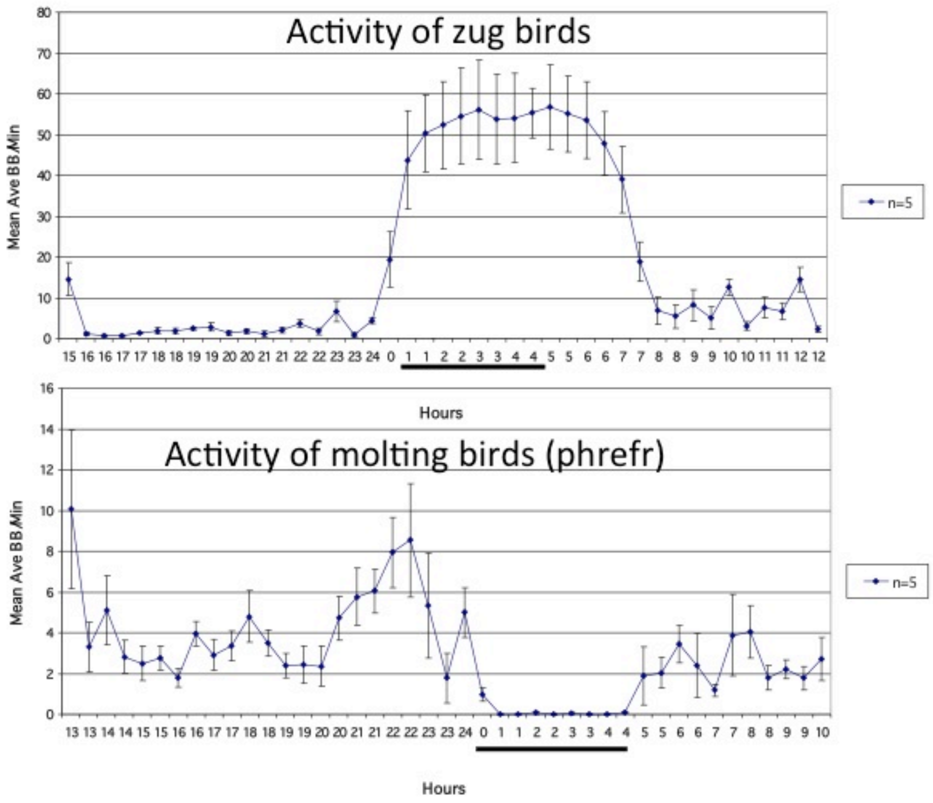

Figure S.4. Perch hopping activity of white-crowned sparrows decreases when given intra-cerebroventricular infusions of 7α-hydroxypregnenolone versus saline as control (top panel). However, a subsequent study reveals no effect of a high dose (hi) or low dose (lo) compared with saline or no infusion (lower panel).

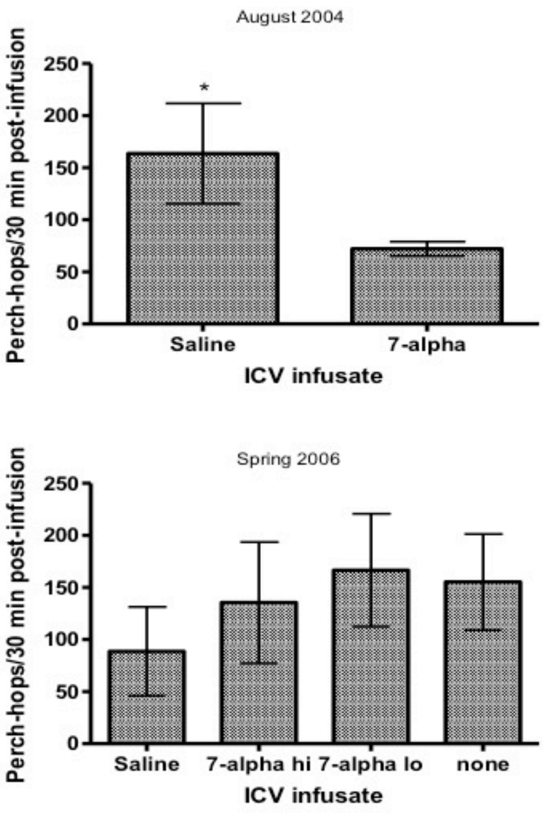

Supplement: Supplementary file 1 [file presentation_1.PDF]
